# Fabrication of Pascal‐triangle Lattice of Proteins by Inducing Ligand Strategy

**DOI:** 10.1002/anie.202000771

**Published:** 2020-04-01

**Authors:** Rongying Liu, Zdravko Kochovski, Long Li, Yue‐wen Yin, Jing Yang, Guang Yang, Guoqing Tao, Anqiu Xu, Ensong Zhang, Hong‐ming Ding, Yan Lu, Guosong Chen, Ming Jiang

**Affiliations:** ^1^ The State Key Laboratory of Molecular Engineering of Polymers and Department of Macromolecular Science Fudan University Shanghai 200433 China; ^2^ Institute of Electrochemical Energy Storage Helmholtz-Zentrum Berlin für Materialien und Energie 14109 Berlin Germany; ^3^ Center for Soft Condensed Matter Physics and Interdisciplinary Research School of Physical Science and Technology Soochow University Suzhou 215006 China; ^4^ Institute of Chemistry University of Potsdam 14476 Potsdam Germany; ^5^ Multiscale Research Institute of Complex Systems Fudan University Shanghai 200433 China

**Keywords:** inducing ligand, pascal triangle, self-assembly, wheat germ agglutinin

## Abstract

A protein Pascal triangle has been constructed as new type of supramolecular architecture by using the inducing ligand strategy that we previously developed for protein assemblies. Although mathematical studies on this famous geometry have a long history, no work on such Pascal triangles fabricated from native proteins has been reported so far due to their structural complexity. In this work, by carefully tuning the specific interactions between the native protein building block WGA and the inducing ligand **R‐SL**, a 2D Pascal‐triangle lattice with three types of triangular voids has been assembled. Moreover, a 3D crystal structure was obtained based on the 2D Pascal triangles. The distinctive carbohydrate binding sites of WGA and the intralayer as well as interlayer dimerization of RhB was the key to facilitate nanofabrication in solution. This strategy may be applied to prepare and explore various sophisticated assemblies based on native proteins.

## Graphical abstract

**A Pascal‐triangle lattice** is constructed by using a careful selection of protein building blocks with anisotropic shapes and two sets of carbohydrate binding sites. Moreover, the dynamic and exchangeable nature of non‐covalent interactions makes the further manipulation of 2D lattices to 3D crystals possible.

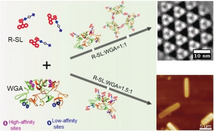

## Introduction

Fractal geometries such as the Sierpinski triangle,^1^ tree‐like architectures,^2^ and other self‐similar entities^3^ have attracted increasing attention of chemists due to their importance in mathematics, engineering and life science.^4^ However, the Pascal triangle, as another significant type of pattern which appears similar to the Sierpinski triangle but is not fractal, has rarely been considered by chemists. Virtually, the Pascal triangle, to some extent, can be regarded as origin of the Sierpinski triangle.^5^ In‐depth investigating on Pascal triangles not only provides access to further ascertain the origin of fractals but also holds great potential for gaining a coherent description of the design principles underlying living organisms.^6^ However, the study of Pascal triangles so far is still limited to the field of mathemathics as a consequence of its high complexity; that is, it still remains a great challenge to fabricate Pascal‐triangle entities via self‐assembly strategies.

Recently, through protein self‐assembly, lots of nanostructures with high complexity have been obtained,^7^ including hollow tubes,^8^ ring‐like structures,^9^ multiscale layers and crystals,^10^ which fully exhibited the high capability of protein self‐assembly to construct sophisticated nano‐sized objects. We proposed and developed a strategy to construct protein assemblies through dual non‐covalent interactions mediated by a small molecular “inducing ligand”, connecting lectins have to form nanoscaled architectures by carbohydrate‐protein interactions and dimerization of rhodamine B (RhB).^11^ However, to the best of our knowledge, biomacromolecular Pascal triangles have not been achieved by using native proteins as building blocks. In our previous work,^11^ the utilization of identical carbohydrate‐binding sites resulted in the formation of isotropic protein self‐assemblies. The key to the construction of Pascal triangles seems to be distinctive multiple interactions between building blocks. Hence, proteins with distinctive carbohydrate binding sites, rather than identical carbohydrate‐binding sites as we previously employed, could be suitable for the construction of Pascal triangles. In this paper, we investigate the utilization of native proteins for the fabrication of Pascal triangles through self‐assembly. We investigated the assembly of wheat germ agglutinin (WGA) with sialyllactoside‐linked RhB as the small molecular ligand; the unique nature of eight carbohydrate‐binding sites of WGA should facilitate specific binding between WGA. Through controlling the ratio of inducing ligand to WGA, Pascal‐triangle 2D lattices with three types of triangular voids and 3D crystals of WGA were constructed. To the best of our knowledge, such fabrication of Pascal‐triangle 2D lattices from native proteins has not been reported in the literature. The results reported here demonstrate great potential of the inducing ligand strategy for construction of protein Pascal triangle patterns with high complexity and provide in‐depth understanding of the design principles of sophisticated geometries in living organisms.

## Results and Discussion

### Choices of Protein Building Block and Inducing Ligand

Given that the Pascal triangle shares similar construction rules with the Sierpinski triangle, two critical rules for the construction of the latter have to be considered:^1^ (1) Building blocks with anisotropic shapes are favored (V‐shape or K‐shape). (2) The optimal selection of driving force is a combination of two or more interactions with different strength. The strong interaction is likely to be responsible for initially assembling the small molecules into basic building blocks, and then the weak one tends to enable the basic building blocks to further assemble into a Sierpinski triangle. According to these design rules, both the shape and carbohydrate‐binding sites of the protein used to construct a Pascal triangle are preferred to be anisotropic.

**Figure 1:**
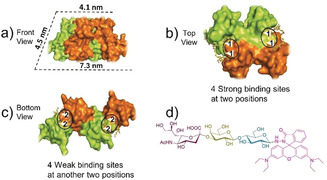
a) The shape of a WGA dimer, one monomer was colored with orange and another monomer was shown as yellow green (structure adapted from reported crystal structure, PDB code: 2X52). b) The 4 strong carbohydrate‐binding sites at two positions (white triangle, four strong carbohydrate‐binding sites were labeled as “1” on the top of WGA). c) The 4 weak carbohydrate‐binding sites at another two positions (white triangle, labeled as “2” at the bottom of WGA). The four black circles in b and c represent the four distribution positions of binding sites. d) The molecular structure of inducing ligand (**R‐SL**).

After careful selection, it was found that wheat germ agglutinin (WGA) appears to be an ideal candidate for construction of a Pascal triangle. WGA is a homodimer, with each monomer containing four domains. The two monomers dimerize in a “head‐to‐tail” fashion, forming a horseshoe‐shaped complex12a (Figure 1 a). This anisotropic shape seems to favor the Pascal triangle construction. Meanwhile, eight independent carbohydrate‐binding sites are concentrated at four positions on the monomer interface of WGA. The four binding sites on the top of WGA (Figure 1 b) exhibit a higher affinity than those at the bottom (Figure 1 c), with a stronger binding constant of at least a factor of two.^12^ In terms of interactions, the four high‐affinity carbohydrate‐binding sites on the top of WGA are roughly identical, and the same is the case for the four low‐affinity ones. Therefore, there are two sets of carbohydrate‐binding sites: four strong binding sites distributed at two positions present on the top of WGA (labeled as 1 in Figure 1 b) and four weak binding sites distributed at another two positions located at the bottom of WGA (labeled as 2 in Figure 1 c).12a, 12c The isothermal titration calorimetry (ITC) experimentally indicated the presence of two sets of binding sites on a WGA (Figure S1 in the Supporting Information).

**Figure 2:**
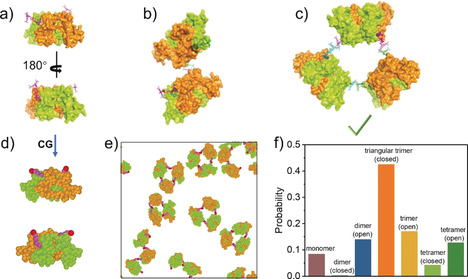
The results of molecular dynamics (MD) simulations and Brownian dynamics (BD) simulations. a) The all‐atom MD result of two ligands binding to the strong sites of WGA protein. The possible packing way of b) two WGA proteins and c) three WGA proteins via RhB dimerization. d) Schematic illustration of the coarse‐grained (CG) model for the WGA proteins (with two ligands at the strong binding sites). e) The final snapshot for the assembly of twenty‐five proteins in BD simulation. f) The probability of different states of proteins in the simulations (averaged by seven independent runs, i.e., Figure 2 e and Figure S7a–f). Open trimer refers to trimer with non‐closed geometry, while closed trimer means those trimers with closed‐loop geometry. For dimer and tetramer, details are shown in Figure S7.

To fabricate a Pascal‐triangle 2D lattice, WGA is supposed to be capable of initially forming a triangular basic building block after addition of the inducing ligand. Based on our previously reported strategy,^11^ we here synthesized an inducing ligand composed of two parts: α2,3‐sialyllactose, which is responsible for binding with WGA; RhB, whose dimerization connects the ligand‐attached WGA. Here this inducing ligand was denoted as **R‐SL** (Figure 1 d). The all‐atom molecular dynamics (MD) simulation was firstly employed to examine whether WGA can form a triangular complex after binding with **R‐SL**. Given that the strong carbohydrate‐binding sites distributed at two positions would be firstly occupied and the steric effect (detailed discussion will be given in the next part) could arise from pre‐binding **R‐SL** at one position, the WGA attached by two **R‐SL** was firstly used in a simulation. After a 20‐ns equilibrium, the final orientation of two **R‐SL** was not parallel and their relative orientation to the protein was also different (Figure 2 a). Such a property may not favor the formation of a closed dimer but makes triangular trimer construction possible (Figure 2 b,c).

Aiming at further estimating the probability of WGA forming triangular trimers, coarse‐grained (CG) Brownian dynamics (BD) simulation was applied, where each amino acid was treated as one CG bead in the simulation (Figure 2 d). Initially, 25 WGA proteins containing two **R‐SL** (on the strong sites) were placed in the simulation box (Figure S2). As time proceeded, the triangular trimer firstly appeared at 43 800 τ (Figure S3c), then more and more triangular trimers can be found in the simulation box (Figure 2 e,f, S3e). However, here the closed dimer was not observed during the whole simulation period (Figure 2 e,f), which was consistent with the result from all‐atom MD simulations. Besides, we also observed closed tetramers in the simulation, but it was a rare event, because this type of closing loop would sharply decrease with increased monomers (Figure 2 f, S7). In addition, due to the limited time scale of the simulation, some non‐closed oligomers such as open dimers, trimers and tetramers still remained (Figure S7). Whereas due to their flexibility, these oligomers were not able to further assemble into large and ordered structures. In general, these simulation results suggested the feasibility of our design, namely, WGA could initially form triangular trimers, which would be used as basic building block in the subsequent Pascal‐triangle 2D lattice construction.

### Preparation and Characterization of the Pascal‐Triangle 2D Lattice of WGA

**Figure 3:**
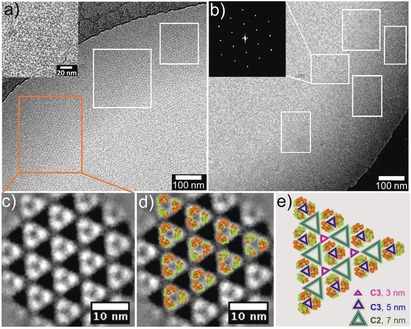
a,b) Cryo‐EM image of the WGA 2D lattice (inset of a: enlarged Cryo‐EM image of the WGA 2D lattice; inset of b: Fourier‐transform of selected area). c) 2D class average of 414 sub‐areas from Cryo‐EM images of the 2D lattice (one white dot represents one WGA protein dimer). d) Overlay of WGA crystal structures (PDB:2X52) with the 2D class average. e) Pascal‐ triangle pattern model (three types Pascal triangle pattern coexist, containing: small triangle void (pink, C3 symmetry, 3 nm length side), medium triangle void (blue, C3 symmetry, 5 nm length side), big triangle void (green, C2 symmetry, 7 nm length side)).

The corresponding synthesis details for **R‐SL** are shown in the Supporting Information (Scheme S1). The procedure of generating a 2D protein lattice is as follows: equal molar concentrations of **R‐SL** and WGA were mixed in 4‐(2‐hydroxyethyl)‐1‐piperazineethanesulfonic acid (HEPES) buffer ([HEPES]=20 mm, [NaCl]=40 mm, [CaCl_2_]=5 mm); the final concentration of **R‐SL** and WGA was 2.0×10^−4^ m. The mixture was incubated at 4 °C for 48 h, then the Pascal‐triangle 2D lattice was obtained as observed under Cryo‐EM. The results shown in Figure 3 a and Figure S4 demonstrate a clear lattice structure, with representative areas marked with white boxes. It can be clearly seen that a triangle structure, rather than the trapezoid shape of WGA, constituted the 2D lattice (Figure 3 a inset). The self‐assembly of WGA induced by **R‐SL** was also confirmed by dynamic light scattering (DLS) (Figure S5). The height of this 2D lattice was about 4.1 nm as revealed by atomic force microscopy (AFM) (Figure S6), which was consistent with the width of WGA, indicating that the 2D lattice was mono‐layered.

The successful construction of the Pascal‐triangle 2D lattice was further confirmed by reference‐free 2D class averaging shown in Figure 3 c,d. Fourier transformation suggested the crystal nature of this 2D lattice (Figure 3 b inset). In addition, it was notable that planar crystals of such large size could grow without support within the confines and be dispersed in solution, which was supported by DLS data (Figure S8).

To identify the driving force of the formation of the Pascal‐triangle 2D lattice, several control experiments have been conducted. Firstly, DLS measurement showed that the WGA was not able to self‐assemble into such a 2D lattice in the absence of **R‐SL** (Figure S9), which was consistent with previous literature.^13^ Secondly, UV/Vis and circular dichroism (CD) spectra results confirmed the RhB dimerization between **R‐SL** (Figure S10). The red‐shift in the UV/Vis absorption spectrum could be ascribed to the dimerization of RhB. Meanwhile, the obvious CD signal is in good agreement with the peak found in UV/Vis, further confirming the occurrence of RhB dimerization in the chiral environment provided by the proteins. Moreover, the addition of either free α2,3‐sialyllactose, which competitively bound with WGA; or β‐CD, which inhibited the dimerization of RhB, leads to the dissociation of the assemblies (Figure S11). This again confirmed that the formation of such Pascal‐triangle 2D lattice was mainly driven by the dimerization of RhB.

We used cryo‐EM to trace the formation process of the Pascal‐triangle 2D lattice. Some triangular aggregates with about 7 nm side length were firstly observed after incubation for 8 h (Figure S12a). When increasing the incubation time to 16 h, clusters composed of three triangular aggregates were also detected (Figure S12b). In addition, the DLS analysis confirmed the sequential formation from triangular trimers to clusters (Figure S8). In short, the self‐assembly process observed here was consistent to our design.

How did the triangular trimers form and further assemble into a Pascal‐triangle 2D lattice? Given the presence of two sets of binding sites on a WGA (Figure S1) and the proximity of two binding sites at each position (Figure 1 b,c), the steered all‐atom molecular dynamics (MD) simulation was employed to test the probability of the ligand binding to the different sites, where one free ligand was pulled to the strong binding site of the WGA with a slow velocity (≈0.2 nm ns^−1^) in the absence (case 1) and presence (case 2) of the adjacent pre‐binding ligand, and to the weak binding site in the absence (case 3) and presence (case 4) of the adjacent pre‐binding ligand, respectively (Figure S13a). As shown in Figure S13b, the pulling forces exhibited a sharp decrease when the ligand began to pack into the binding site in case 1 and case 3, but the decrease in case 1 was more sharp than that in case 3, suggesting that it was easier for the ligand to be occupied to the strong binding site. More importantly, the pulling forces both increased monotonously in case 2 and case 4, possibly because of the steric effect between the free ligand and the pre‐binding ligand (Figure S13c, d). As a result, the probability of the ligand binding to the strong site with adjacent pre‐binding ligand (i.e., case 2) became lower than that of the ligand binding to the weak site without adjacent pre‐binding ligand. Moreover, we also compared the binding energy of the WGA and the ligand, and the binding energy in case 1 was the lowest among the four cases (Figure S14). On the basis of the discussion above, the order of binding priority appears to be as follows: case 1 > case 3 > case 2 > case 4, namely, two strong binding sites distributed at different positions tended to be firstly occupied, and then two weak binding sites distributed at different positions subsequently were occupied, the remaining two strong binding sites and two weak binding sites having adjacent pre‐binding ligands were finally occupied.

Notably, the experiment and theoretical calculation results suggested that about four ligands bound to each protein as the ratio of inducing ligand **R‐SL**/WGA was set to 1:1 (Figure S15, Table S1). Consequently, based on the discussion above, two of the four ligands would firstly bind to the strong sites distributed at different positions, allowing the formation of triangular trimers. Then another two ligands may occupy two weak binding sites located at different positions, permitting the triangular trimers to assemble into clusters and further into the Pascal‐triangle 2D lattice. On the contrary, when the ratio of **R‐SL** to WGA was decreased to 0.5:1 (in this case, the average number of ligands binding to each protein was about two, see Table S1 and Figure S15), only triangular trimers but no 2D lattice appeared (Figure S16). In other words, the dimerization of RhB at the strong binding sites can only induce the formation of triangular trimers but that at the weak binding sites is essential for the further assembly of WGA into Pascal‐triangle 2D lattice.

**Scheme 1:**
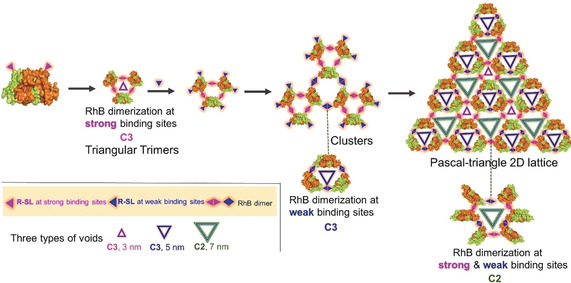
The proposed self‐assembly mechanism underlying the formation of Pascal‐triangle 2D lattice with p3 symmetry. It includes two sets of RhB dimerization (RhB dimerization at strong binding sites results in triangular trimer with C3 symmetry, RhB dimerization at weak binding sites gives rise to trimer with C3 symmetry, combination of two sets of RhB dimerization leads to hexamer with C2 symmetry.

Based on the simulation and experimental results, the mechanism underlying the formation of the Pascal‐triangle 2D lattice was proposed as illustrated in Scheme 1. At first, the RhB dimerization at strong binding sites gave rise to triangular trimers; then the RhB dimerization at weak binding sites tended to trigger the assembly of the triangular trimers into clusters and further into a Pascal‐triangle 2D lattice.

More interestingly, along with WGA assembled into a Pascal‐triangle pattern with p3 symmetry, three types of equilateral triangle voids were also formed. Three WGAs made up equilateral triangle voids with 3 nm side‐length and C3 symmetry by RhB dimerization at strong binding sites. Similarly, equilateral triangle voids with 5 nm side‐length, C3 symmetry by RhB dimerization at weak binding sites, and six WGAs made up equilateral triangle voids with 7 nm side‐length. C2 symmetry results from RhB dimerization at both strong and weak binding sites (Figure 3 e, Scheme 1).

Although some transmembrane proteins can self‐assemble into a 2D lattice in a membrane environment, they exist as the free‐monomer state in solution.^14^ Additionally, some researchers found that a 2D lattice could be generated by designed protein interacting interface.7h, 15 However, we checked all the 200 types (up to now) of 2D lattices from Reticular Chemistry Structure Resource (RCSR),^16^ no similar Pascal‐triangle 2D lattice with three types of voids could be found.

### Fabrication and Characterization of 3D Crystals

After elucidating the self‐assembly mechanism of the Pascal‐triangle 2D lattice, we next investigated whether the 2D lattice could be further packed into 3D crystals. As an important driving force of protein self‐assembly, protein‐protein interaction was firstly considered to promote the stacking of 2D lattices. Thus, we attempted to extend the incubation time. However, as the ratio of **R‐SL**/WGA was set to 1:1, no obvious 3D crystals emerged, even after incubation for 120 h (Figure S17). More NaCl was added to the assembly solution to enhance the protein aggregation tendency, but only a few small 3D crystals could be observed (Figure S18). This suggested that here the protein‐protein interaction could, to some extent, promote the stacking of a 2D lattice but was not strong enough to generate large 3D crystals. To strengthen the interaction between layers, given the carbohydrate‐binding sites of WGA were still unsaturated as the ratio of **R‐SL** to WGA was set to 1:1, increasing this ratio may be a practical way to permit more binding sites to be occupied (or even become saturated).11a, 11d, 17 In addition, it was reported that the interlayer dimerization of RhB tended to promote the 2D lattice stacking and 3D crystal growth.^18^ Therefore, it can be envisioned that more inducing ligands may give rise to the stacking of a 2D lattice and 3D crystal growth.

To test this possibility, the ratio of **R‐SL** to WGA was directly increased to 1.5:1. CD and Cryo‐EM were firstly employed to trace the assembly process. During the initial 6 h of incubation, a pronounced increase of the RhB dimerization signal was observed in CD spectra (Figure S19), which often appeared as the RhB dimerization transformed from a free state into a confined state.^18^ Indeed, as observed by Cryo‐EM, some small 3D aggregates appeared after 6 h incubation, despite many Pascal triangles remained (Figure S20a). This indicated that more inducing ligands participated in interlayer RhB dimerization, promoting the growth of 3D aggregates.^18^ When increasing the incubation time to 12 h, the CD signal assigned to RhB dimerization increased significantly (Figure S19), indicating that most of the Pascal triangles assembled into larger 3D aggregates (Figure S20b). Therefore, it can be deduced that more inducing ligands gave rise to interlayer RhB dimerization compared to the case of the 2D lattice, which enabled the formation of 3D aggregates.

**Figure 4:**
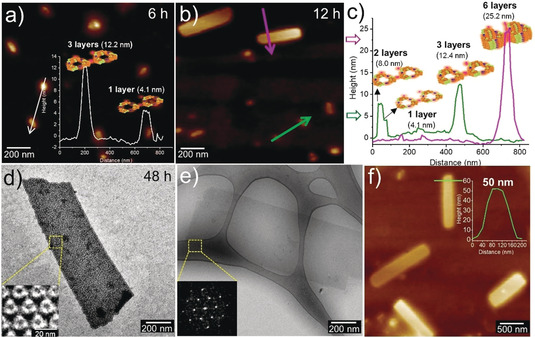
a) the AFM height image of **R‐SL**/WGA (1.5:1) after incubation 6 h. b) The AFM height image and c) corresponding height profiles of (**R‐SL**/WGA (1.5:1) after incubation 12 h. d) Negative stain TEM (inset: 2D class average image of selected area), e) Cryo‐EM (inset: Fourier‐transform of selected area) and f) AFM height image of 3D crystals (**R‐SL**/WGA (1.5:1) after incubation 48 h.

AFM allowed further detection of the assembly process. After incubation of 6 h, both single‐layer 2D lattice and multi‐layer small aggregates were observed (Figure 4 a). Upon increasing the incubation time to 12 h, some larger aggregates appeared (Figure 4 b). A common feature shared by these aggregates was that their thicknesses were integral multiples of that of a single‐layer 2D lattice (4.1 nm). Typically, even in the same aggregate, there were two different thicknesses: 4.1 nm and 8.0 nm (Figure 4 c), implying that certain parts of this aggregate already grown in the third dimension, while the other areas still retained a single‐layer structure. In other words, a hierarchical self‐assembly occurred in two and three dimensions simultaneously.

Notably, regular 3D crystals appeared after 48 h incubation (Figure 4 d,e). The AFM height image suggested that the thickness of 3D crystals was about 50 nm (Figure 4 f), which corresponded to about twelve layers of WGA. Significantly, both the results from 2D class average and Fourier‐transform for this 3D crystal (Figure 4 d inset, Figure 4 e inset) were similar to those of 2D lattices (Figure 3 c,b inset), suggesting that the 3D crystals shared analogous protein packing with 2D lattices.

To summarize, a possible self‐assembly mechanism of 3D crystals was proposed as illustrated in Figure S21. Initially, the WGA still assembled into triangular trimers through RhB dimerization at strong binding sites. Then, the RhB dimerization at weak binding sites enabled that triangular trimers assemble into 2D clusters; at the same time, the interlayer RhB dimerization contributed by more inducing ligands allowed the clusters to further grow in the third dimension, namely, more inducing ligands permitted the simultaneous occurrence of self‐assembly in two dimensions and three dimensions. As a result, these clusters gradually evolved into 3D crystals through self‐assembly.

## Conclusion

In this work, we constructed Pascal‐triangle 2D lattices using well‐selected protein building blocks with anisotropic shapes and two sets of carbohydrate binding sites. Moreover, the dynamic and exchangeable nature of non‐covalent interactions make the further manipulation on 2D lattices to 3D crystals possible. Such results not only demonstrate the first construction of a Pascal‐triangle 2D lattice from native proteins, but also drive the protein lattices fabricated by our inducing ligand strategy to an unprecedented level. We believe that this work opens a new avenue on fabrication of novel protein assemblies with desired shapes, sizes and functionalities, which might help us to understand the design principles underlying living organism better.

## Conflict of interest

The authors declare no conflict of interest.

## Supporting information

As a service to our authors and readers, this journal provides supporting information supplied by the authors. Such materials are peer reviewed and may be re‐organized for online delivery, but are not copy‐edited or typeset. Technical support issues arising from supporting information (other than missing files) should be addressed to the authors.

Supplementary
