# Supplementary material for: Fabrication of Pascal‐triangle Lattice of Proteins by Inducing Ligand Strategy
Source: Angew Chem Int Ed Engl. 2020 Apr 1;59(24):9617–23. doi: 10.1002/anie.202000771 (PMC7318223; doi:10.1002/anie.202000771)
Supplement: Supplementary file 1 — Supplementary [file ANIE-59-9617-s001.pdf]

Angewandte Chemie
Eine Zeitschrift der Gesellschaft Deutscher Chemiker

Supporting Information

Fabrication of Pascal-triangle Lattice of Proteins by Inducing Ligand Strategy

Rongying Liu, Zdravko Kochovski, Long Li, Yue-wen Yin, Jing Yang, Guang Yang, Guoqing Tao, Anqiu Xu, Ensong Zhang, Hong-ming Ding, Yan Lu,* Guosong Chen,* and Ming Jiang*

anie_202000771_sm_miscellaneous_information.pdf

Table of Contents

1. General information S3

2. Two sets of binding sites supported by ITC data S5

3. The all-atom molecular dynamics (MD) simulation and coarse-grained (CG) Brownian dynamics (BD) simulation S6

4. The synthetic procedure of **R-SL** S9

5.Characterization of Pascal triangle 2D lattice S10

6. Circular dichroism (CD) S13

7. Cryo-EM observations of self-assembly process of Pascal triangle 2D lattice S14

8. Steric effect between pre-binding ligand and free ligand as well as binding priority based on MD simulation. S15

9. Calculation results of possible occupied carbohydrate-binding sites of WGA at different ratios of **R-SL** to WGA S17

10. The distribution of **R-SL** between solution and assemblies S19

11. The self-assembly behavior of **R-SL**/WGA = 0.5:1 S20

12. Protein-protein interaction S21

13. Characterization of 3D crystals S22

14. ^{1}H and ^{13}C NMR of **R-SL** S24

1. General information

Sample preparation

The inducing ligand **R-SL** was synthesized and characterized as described in supporting information (Scheme S1 and Figure S28-32). WGA protein was purchased from vector laboratories. All chemicals and proteins are used as received. The buffer solution was prepared with HEPES {4-(2-hydroxyethyl)-1-piperazineethanesulfonic acid} buffer containing 20 mM HEPES, 5 mM $CaCl_2$, and 40 mM NaCl. The WGA/**R-SL** mixture was prepared by mixing the WGA solution and **R-SL** solution together. The WGA solution was prepared by dissolving WGA (lyophilized powder) in buffer and was stored over 2 h at 5 °C. **R-SL** was also dissolved in buffer separately. The solutions were filtered through a Millipore 0.45 μm membrane before mixing.

Characterization

Nuclear magnetic resonance (NMR) was taken by AVANCE III HD 400 MHz of Bruker BioSpin International. Matrix Assisted Laser Desorption Ionization-Time of Flight (Maldi-TOF) Mass Spectrum was taken by a AB SCIEX 5800 instrument. Ultraviolet–vis (UV-vis) absorption spectra were recorded by Shimadzu UV-2550 spectrophotometer. Circular dichroism (CD) spectra was taken by a JASCO-815 instrument. Isothermal titration calorimetry (ITC) experiments were conducted on a MicroCal VP-ITC system at 20.00 ± 0.01 °C.

Data collection

For the preparation of negatively stained samples, a drop of the mixture solution was applied onto a copper grid and the excess solvent was blotted away. Samples were subsequently stained with 1 wt% uranyl acetate. Samples for Cryo-EM were prepared by applying 4 μL drop of mixture solution to holey carbon grids (Quantifoil R2/1) and plunge-frozen into liquid ethane with a FEI vitrobot Mark IV set at 4 °C and 95% humidity. Vitrified grids were either transferred directly to the microscope cryoholder or stored in liquid nitrogen. All grids were glow-discharged before use.

Cryo-EM and negative stain micrographs were acquired at a number of magnifications on a JEOL JEM-2100 equipped with a 4 k × 4 k CMOS digital camera (TVIPS TemCam-F416), operated at 200 kV and on a Philips CM120 operated at 80 kV.

AFM was operated in air on a Bruker Multimode VIII SPM equipped with a J scanner. Experiments were performed in tapping mode and Peak force QNM mode with NSC11 tip (spring constant 48 $N \cdot m^{-1}$, MikroMasch). Sample (5 μL) was placed on a freshly cleaved mica for AFM test under dry conditions. Sample solution was allowed to adsorb for 5 min and then it was washed gently with 1 mL buffer followed by air drying.

CLSM: Confocal laser-scanning microscopy images were taken from Nikon C2+.

2. Two sets of binding sites supported by ITC data

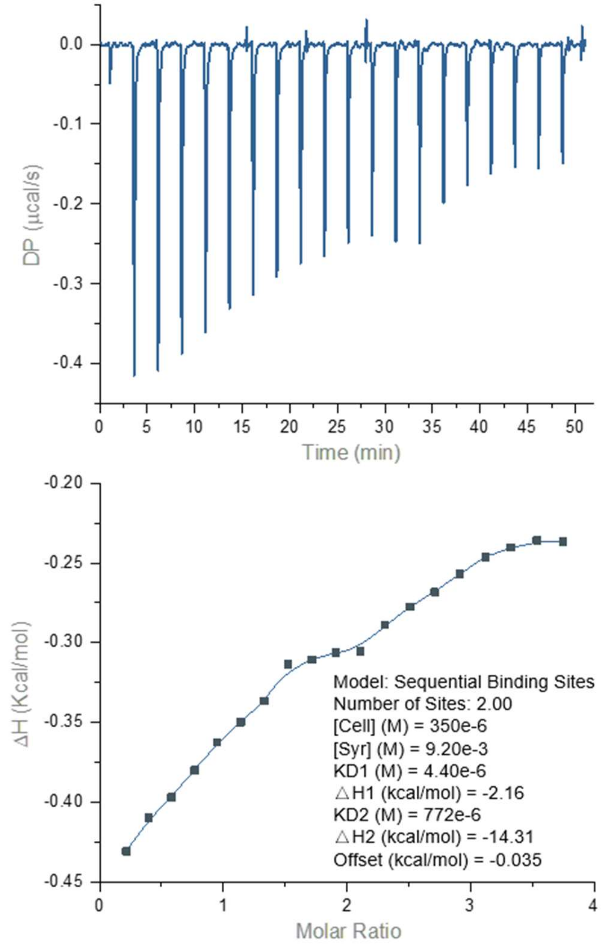

Figure S1. ITC data of titration of 9.2 mM **R-SL** into 0.35 mM WGA at 20 °C in aqueous solution.

3. The all-atom molecular dynamics (MD) simulation and coarse-grained (CG) Brownian dynamics (BD) simulation

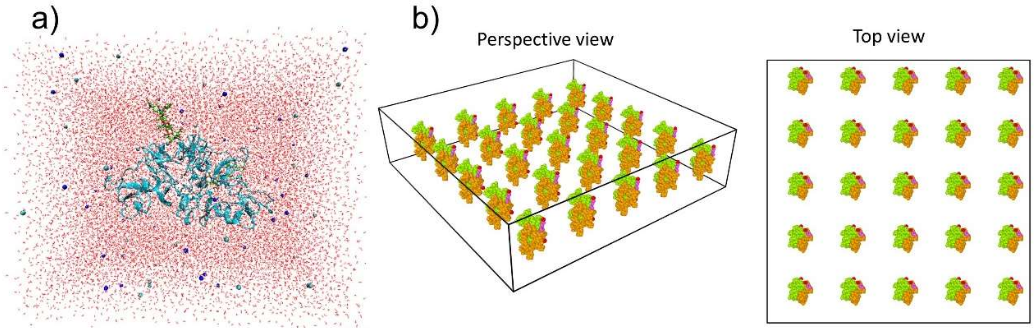

Figure S2. The setup of (a) all-atom MD simulation (one WGA containing two ligands at the strong binding sites) and (b) BD simulation system (twenty-five proteins were uniformly placed in the box at the beginning of the simulation).

As shown in Figure S1a, the all-atom molecular dynamics (MD) simulation system mainly consisted of WGA protein (PDB code: 2X52) and **R-SL** ligand. At the beginning of the simulation, we firstly bind two **R-SLs** to the protein, where the carbohydrate-binding sites were referred from Ref. 1.[1] Then a number of water molecules were added to the system to separate the proteins from their mirrors. The ions (i.e., Na^+, Cl^-) were also added to the simulation box to ensure the electric neutrality of the system.

All all-atom MD simulations were performed by using Gromacs 5.0.4 package[2] with the Amber force field[3] and the TIP3P water model[4] in the NVT ensemble. The force field parameter for **R-SL** was built by using Antechamber tool.[5] During the simulation, the temperature was coupled at 280 K using Nosé-Hoover method.[6-7] The Particle Mesh Ewald (PME) method[8] was used to calculate the electrostatic interactions and the cut-off of Lennard Jones (LJ) interaction was 1.2 nm. The periodic boundary conditions were applied in all three dimensions. The timestep was chosen as 2 fs and each simulation was at least conducted for 20 ns. Further, the MD simulations were repeated three times for each system, starting from independent initial configurations.

In order to the investigate self-assembly process of the WGA proteins at a longer time and length scale, the Brown dynamics (BD) simulations[9] were also used. Actually, since there existed a large number of WGA proteins in this system, it was far beyond

the computing ability of present all-atom molecular simulation. Here, for the sake of simplicity, each amino acid in WGA was represented by a single coarse-grained (CG) bead,[10-11] where the initial coordinate of the CG beads was obtained by using the coordinate of C_{alpha} of the amino acids in all-atom MD simulation. Considering the stability of the main structure of WGA, the relative position of these beads remains the same (i.e., here the WGA protein moved as a rigid body in the BD simulation). Similarly, the **R-SL** ligand was modeled as a polymer with five CG beads. To model the effective RhB dimerization, the last bead of the polymer was specific, and can form dynamic bond with each other.[12] The coating sites of the polymers were also approximately the same as that in all-atom simulations. A shifted Lennard-Jones potential,[13] cut off at $2^{1/6}$ σ, is used to model the repulsive interaction between the protein and polymer beads, where ϵ is chosen as ϵ/kT_0 = 1.0 for all beads, and σ = 1.0 r_0 for protein beads, σ = 0.4 r_0 for polymer beads, σ = 0.7 r_0 for the mixture of polymer beads and protein beads. Additionally, a standard Lennard-Jones potential (cut off at 1.5σ) was used to model the attractive interaction between the specific beads, where ϵ is chosen as ϵ/kT_0 = 5.0 and σ = 0.4 r_0. At the beginning of the simulation, twenty-five proteins were uniformly placed in the box (Fig. S1b).

All BD simulations were performed in the NVT ensemble by using the LAMMPS package (15 May2015).[14] During the simulation, the temperature was coupled at 1.0 T_0 using the Langevin thermostat. The time step was 0.006 τ, and the data were collected every 50 τ, with the total simulation time lager than 300000 τ.

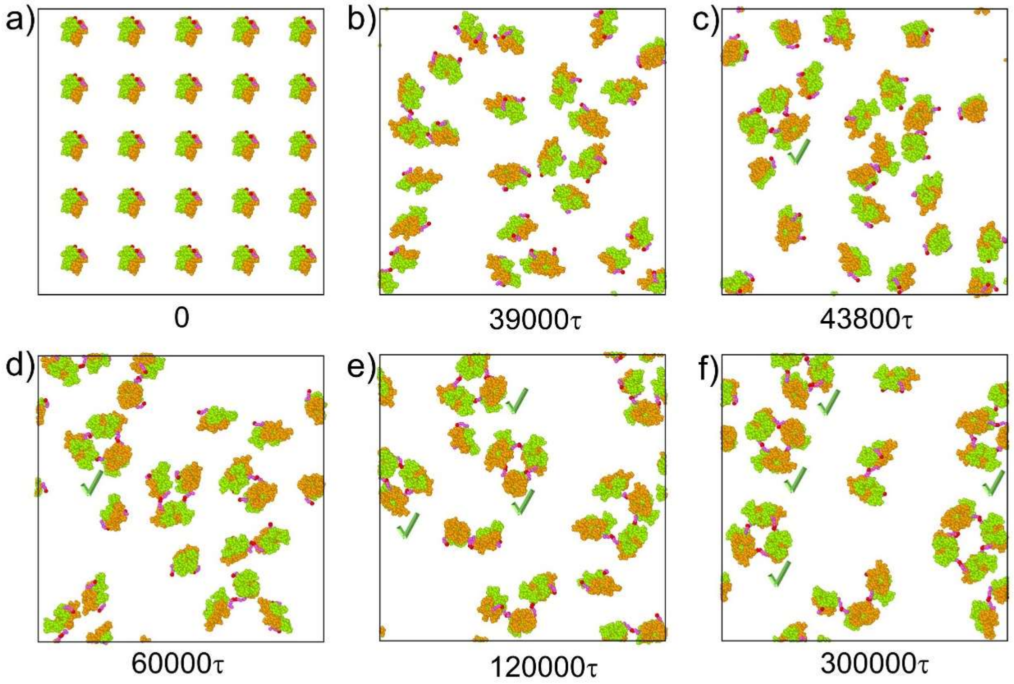

Figure S3. a-f) Time sequence of snapshots illustrating the self-assembly process of the proteins in the BD simulation.

4. The synthetic procedure of R-SL and ITC data

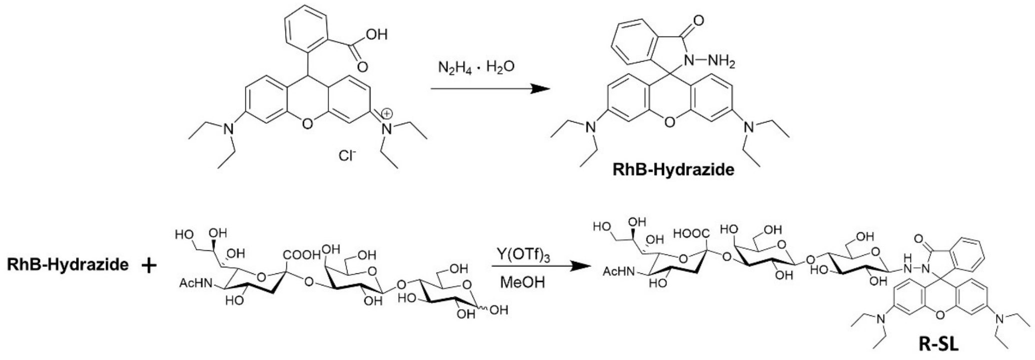

Scheme S1. Synthetic procedure of **R-SL** used in this paper.

5. Characterization of Pascal-triangle 2D lattice

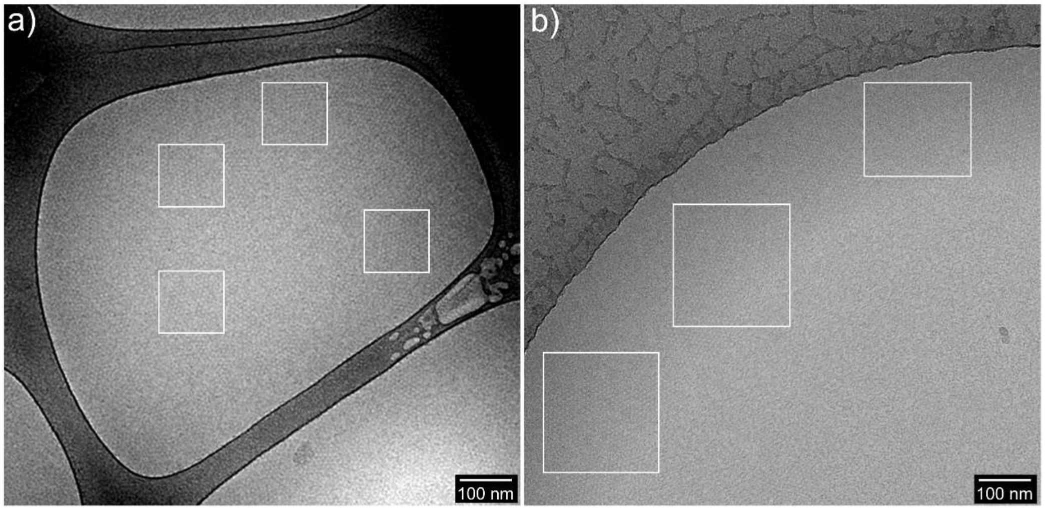

Figure S4. a, b) Cryo-EM images of 2D lattice of **R-SL**/WGA (1:1) after 48 h incubation at 4 °C.

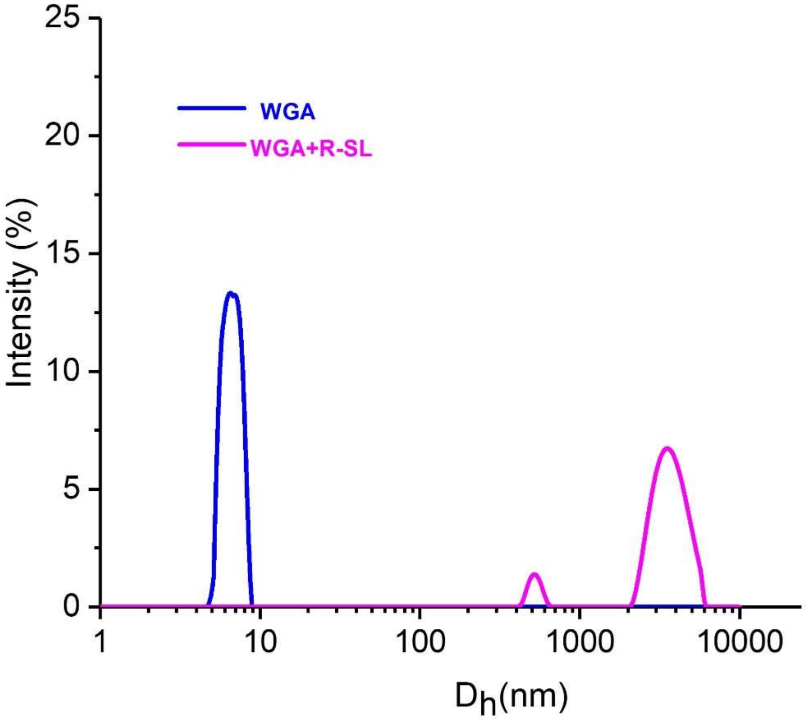

Figure S5. The dynamic light scattering (DLS) results of **R-SL**/ WGA (2.0×10^{-4} M) after incubation for 48 h.

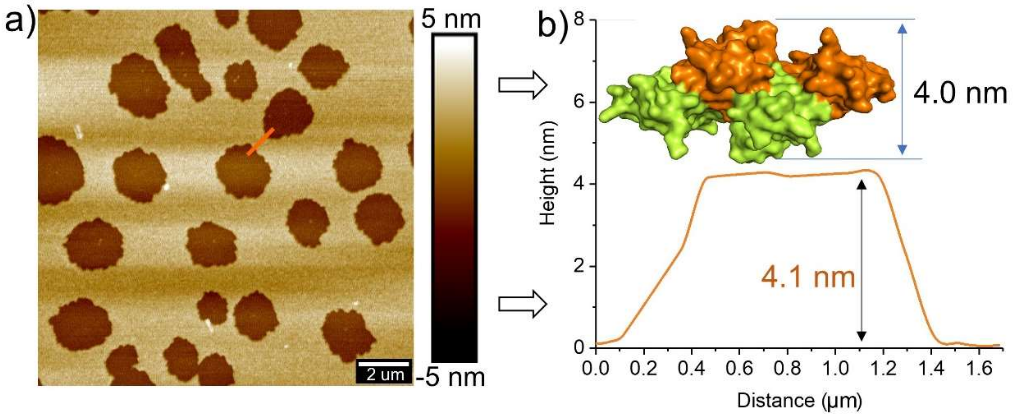

Figure S6. a) The AFM height image b) the corresponding height profile of 2D lattice from the **R-SL** /WGA (1:1) after 48 h incubation.

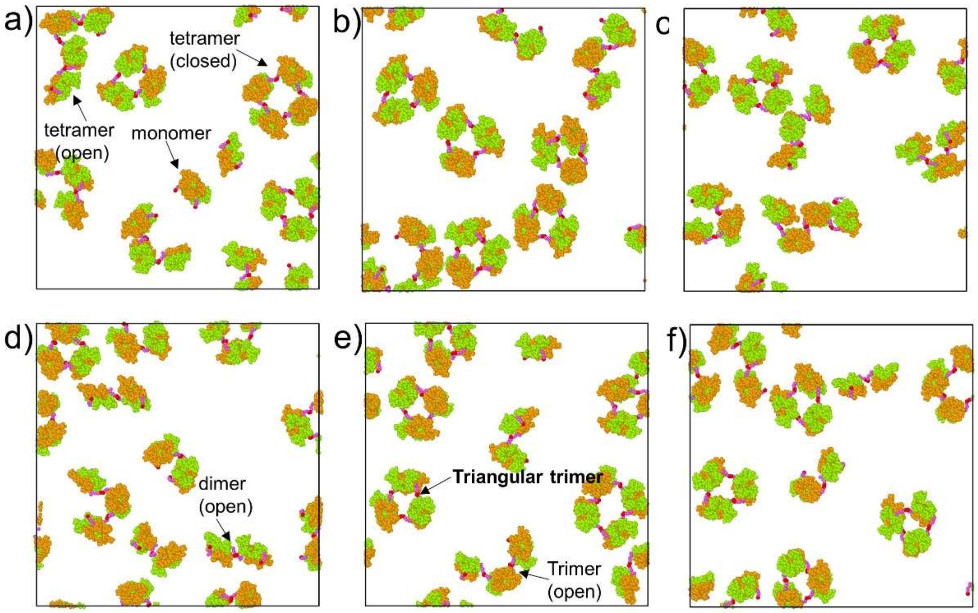

Figure S7. a-f) Six typical final snapshots from six independent runs in the BD simulation.

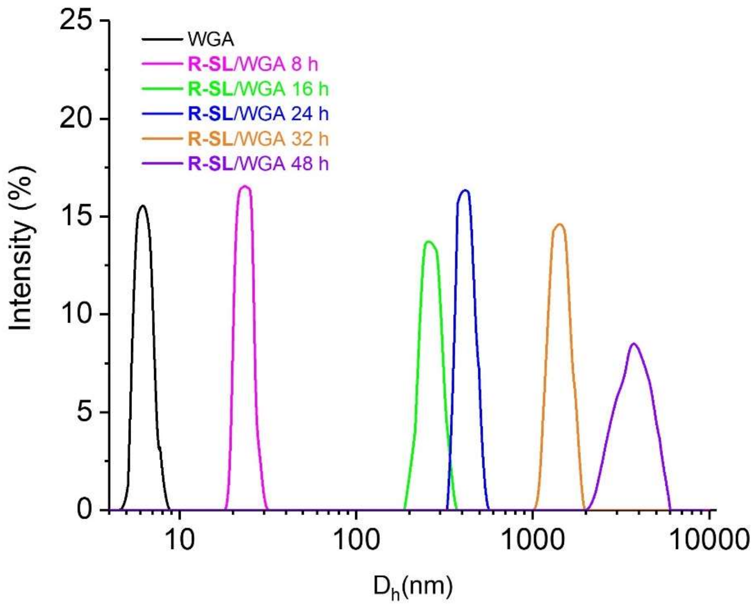

Figure S8. The DLS results of **R-SL**/WGA (1:1) as a function of incubation time.

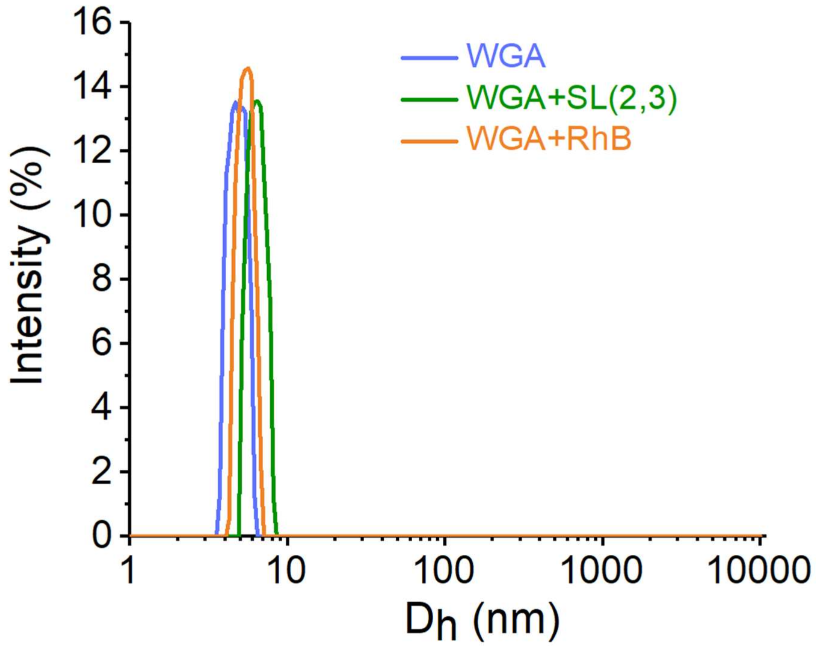

Figure S9. The DLS results of single WGA, WGA+SL (2,3) and WGA+RhB after incubation for 48 h.

6. Circular dichroism (CD)

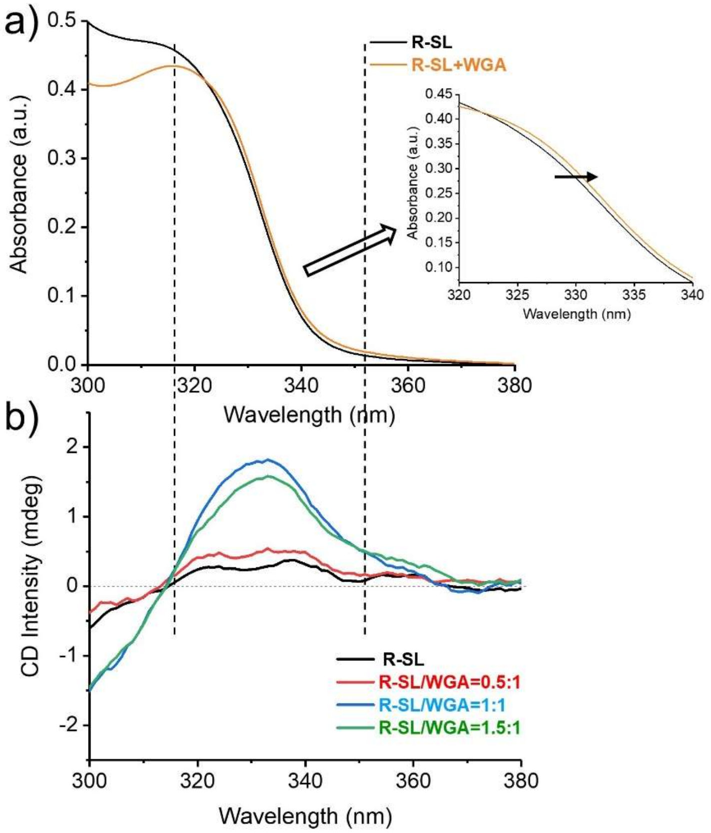

Figure S10. a) The UV-vis absorbance of **R-SL**/WGA (2×10^{-4} M) mixture after incubation for 48 h in aqueous solution at 5 °C (the inset is enlarged UV-vis spectra). b) The Corresponding CD spectrum of **R-SL**/WGA (2×10^{-4} M) mixture of different ratios of **R-SL** to WGA after incubation for 48 h in aqueous solution at 5 °C.

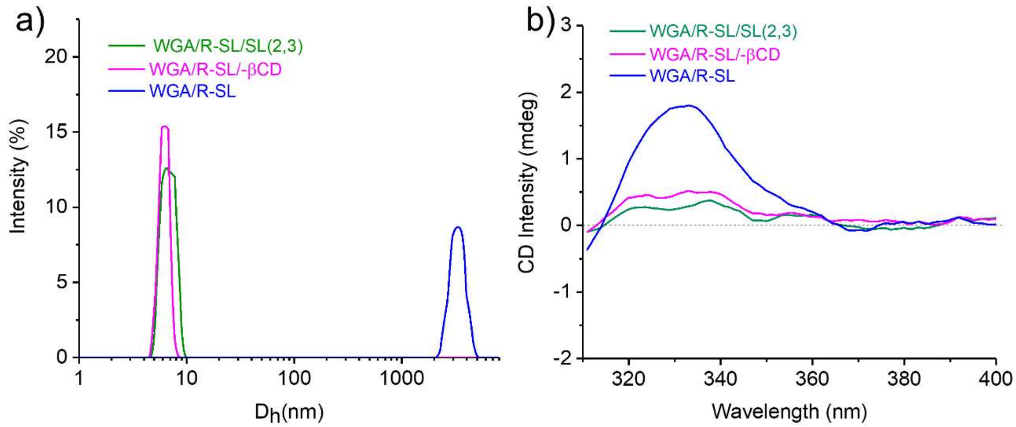

Figure S11. a) DLS and b) CD spectra of **R-SL**/ WGA (1:1) before and after addition of SL(2,3), β-CD.

7. Cryo-EM observations of self-assembly process of Pascal-triangle 2D lattice

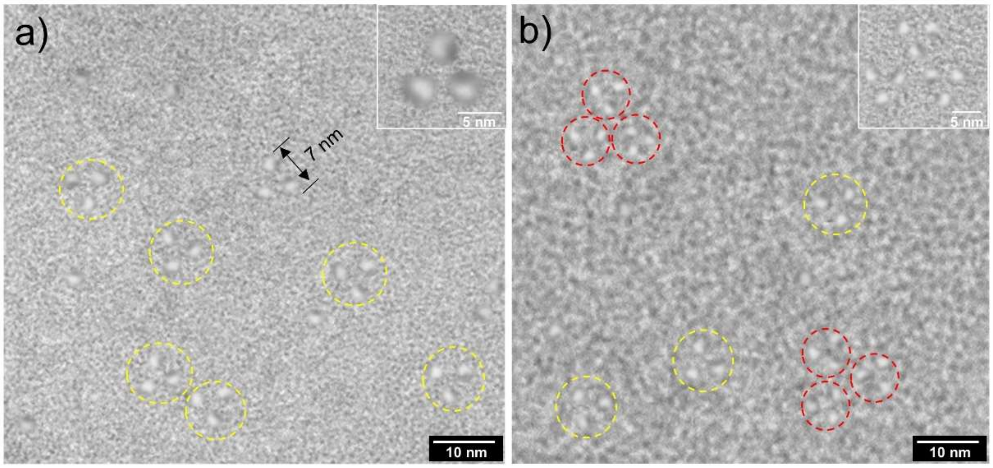

Figure S12. a) the Cryo-EM image of triangular trimer of **R-SL**/ WGA (1:1) after incubation for 8 h. (inset: enlarged image of an individual trimer).
b) the Cryo-EM image of clusters of **R-SL**/WGA (1:1) after incubation for 16 h. (inset: enlarged image of an individual cluster).

8. Steric effect between pre-binding ligand and free ligand as well as binding priority based on MD simulation.

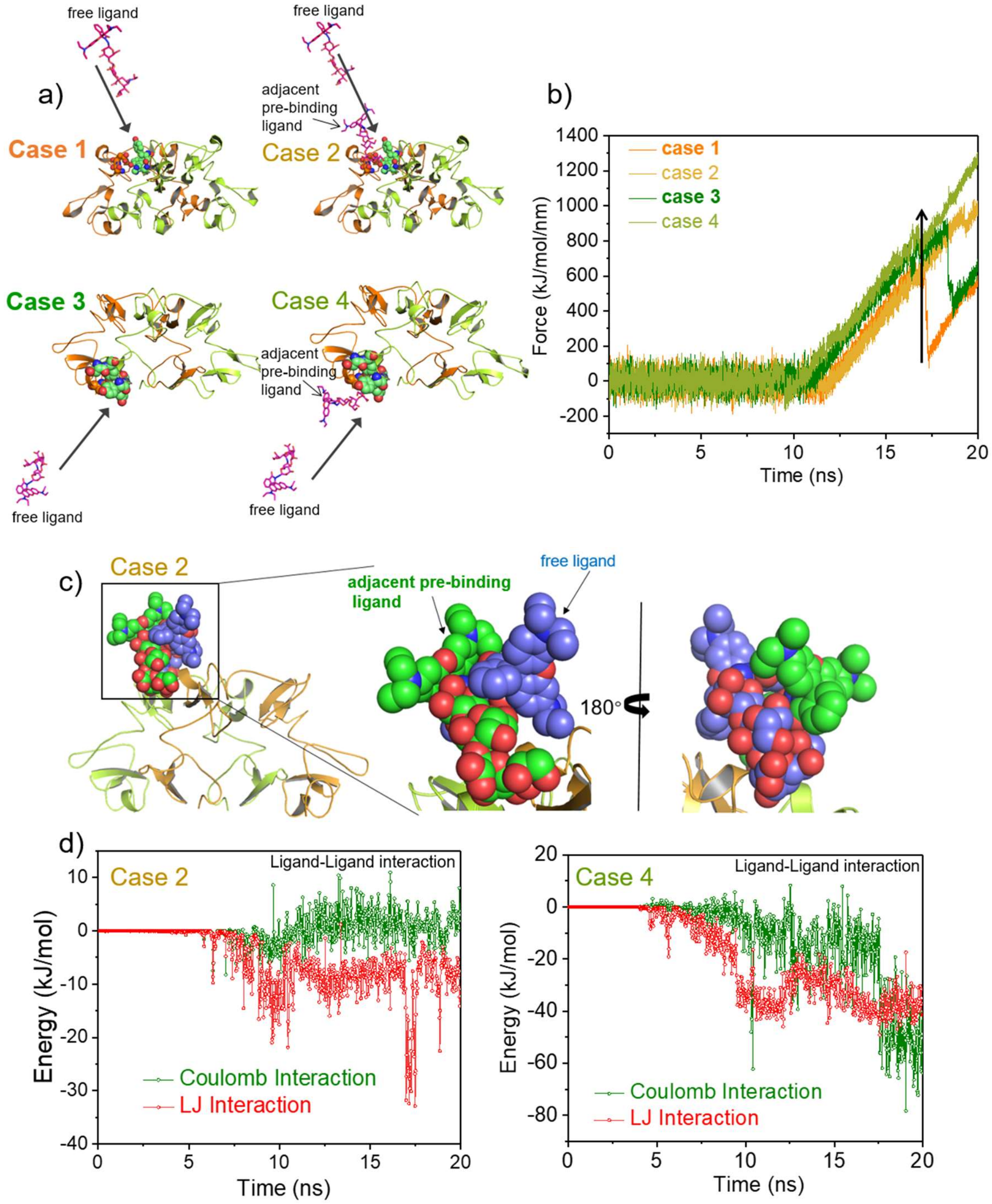

Figure S13. Schematic illustration of one free ligand was pulled to the strong binding site of the WGA with a very slow relative velocity (~0.2 nm/ns) in the absence (case 1) and presence (case 2) of the adjacent pre-binding ligand, and to the weak binding site in the absence (case 3) and presence (case 4) of the adjacent pre-binding ligand (Fig. S2a), respectively.

(a) Cartoon illustration for four cases, (b) The corresponding pulling force profile in different cases, (c) Illustration for steric effect between the pre-binding ligand and the free ligand (take the case 2 as an example), (d) The Lennard-Jones (LJ) and Coulomb interaction energy between ligand and ligand during the simulation in case 2 and case 4.

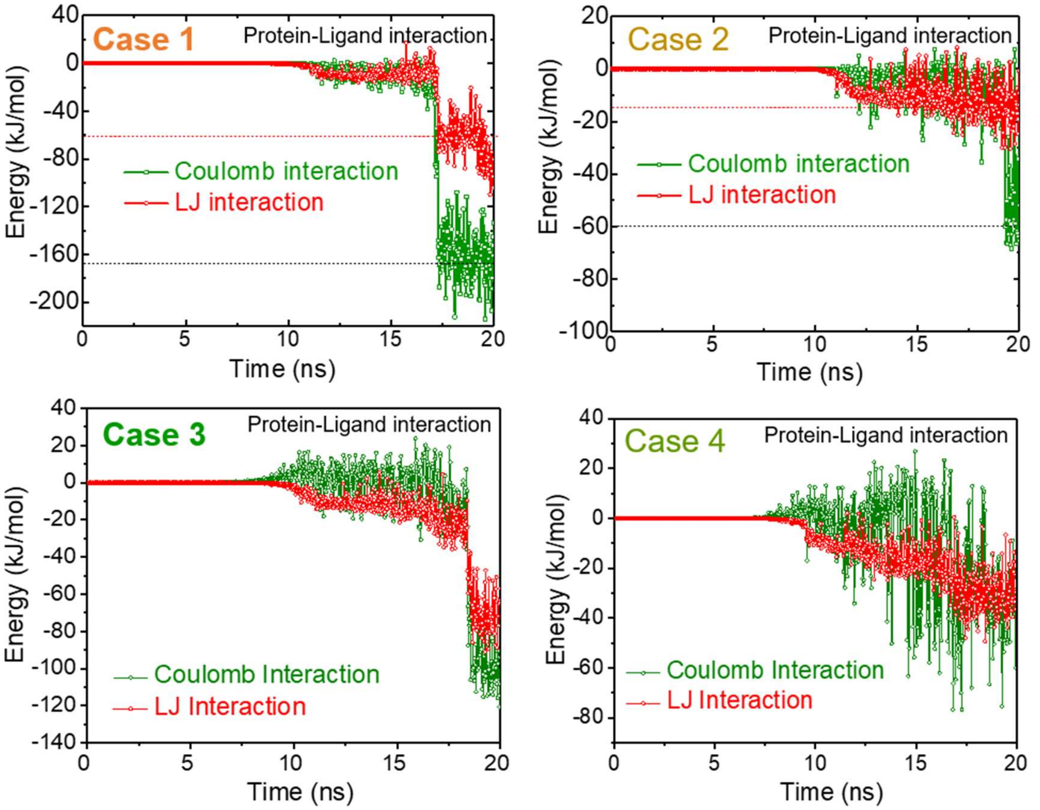

Figure S14. The Lennard-Jones (LJ) and Coulomb interaction energy between WGA and the ligand during the simulation in case1, case 2, case 3 and case 4.

Movie S1(see the attachment): This movie displays how the adjacent pre-binding ligand at one strong binding site prevent the other free ligand from binding to the second binding site at that position

9. Calculation results of possible occupied carbohydrate-binding sites of WGA at different ratios of R-SL to WGA

The broad distribution of **WGA** attached different inducing ligands could be due to relatively weak binding between WGA (denoted as **M**) and Sialyllactose(2,3) of **R-SL** (denoted as **X**), as revealed by the following discussion. Based on ITC technique, for an inducing ligand X binding to a single set of n unique sites on the WGA:

$$M+X=MX$$
$$M+X_2=MX_2$$
$$\cdots\cdots$$
$$\cdots\cdots$$
$$MX_{n-1}+X=MX_n$$

the single site binding constant is defined by ITC experiment as:

$$K=\frac{[\text{filled sites}]}{[\text{empty sites}][X]}$$

Thus, if the binding sites of **M** is denoted as **H**, and the same bulk concentration (denoted as C) of **H** and **X** is used, the fraction of **H** occupied by **X** (denoted as **P**) can be calculated as follows:

$$H+G \rightarrow HX$$

$$K=\frac{[HX]}{[H]\times[X]}=\frac{PC}{(1-P)(N-P)C^2}$$

$$P=\frac{[KC^2(N+1)+1]-\sqrt{[KC^2(N+1)+1]^2-4(KC)(NKC)}}{2KC}$$

For binding between each **WGA** binding site (**H$_1$**) and **R-SL** (X_1), of which the same bulk concentration was fixed as C, K_1 denotes the binding constant and P_1 denotes the fraction of **WGA** binding sites occupied by **R-SL**, which also means the conjugated efficiency of **R-SL** to **WGA**. Similarly, for dimerization among **R-SL**s (X_2) where the bulk concentration denoted as C, K_2 denotes the binding constant, and P_2 denotes the fraction of one R-SL dimerized with adjacent counterpart. Thus:

$$P_1=\frac{[K1C^2(N+1)+1]-\sqrt{[K1C^2(N+1)+1]^2-4(K1C)(NK1C)}}{2K1C}$$

$$P_2=\frac{[K2C^2(N+1)+1]-\sqrt{[K2C^2(N+1)+1]^2-4(K2C)(NK2C)}}{2K2C}$$

Where P_1 also means the possibility of each **WGA** binding sites occupied by **R-SL**, P_2 means the possibility of dimerization of R-SL. Thus, the possibility of each WGA binding site being successful conjugated to adjacent binding site of another WGA can be

$$P' = P_1\times P_2$$

$$=\left\{\frac{[K1C^2(N+1)+1]-\sqrt{[K1C^2(N+1)+1]^2-4(K1C)(NK1C)}}{2K1C}\right\}\times\left\{\frac{[K2C^2(N+1)+1]-\sqrt{[K2C^2(N+1)+1]^2-4(K2C)(NK2C)}}{2K2C}\right\}$$

Which also means the link efficiency among adjacent **R-SL**, thus the average number of R-SL conjugate to each WGA is

$$\bar{P} = 8 \times P'$$

Moreover, considering there are eight carbohydrate-binding sites on each WGA, it may conjugate 0, 1, 2, 3, 4, 5, 6, 7 or 8 R-SL, leading to nine possible states of R-SL conjugation, The fraction of these different states can be denoted as Fn (n = 0-8):

$$Fn= Pn\times(1-P)8-n\times C_8^n$$

So, we can calculate the concentration dependence of Fn as shown in Table S1.

Occupied Carbohydrate-binding sites	Probability		
	0.5：1	1：1	1.5：1
0	0.071824916	0.00533932	6.90348E-05
1	0.224003277	0.039441613	0.001276978
2	**0.305641046**	0.127468074	0.010334196
3	0.238303781	0.235401989	0.047789447
4	0.116126198	**0.271705762**	0.138123423
5	0.036216748	0.200709322	0.255495135
6	0.007059415	0.092665112	**0.295377871**
7	0.000786303	0.024447076	0.195135025
8	0.000038316	0.002821731	0.05639889

Table S1. The possible occupied carbohydrate-binding sites while the ratio of **R-SL**/WGA was set to 0.5:1, 1:1 and 1.5:1.

10. The distribution of R-SL between solution and assemblies

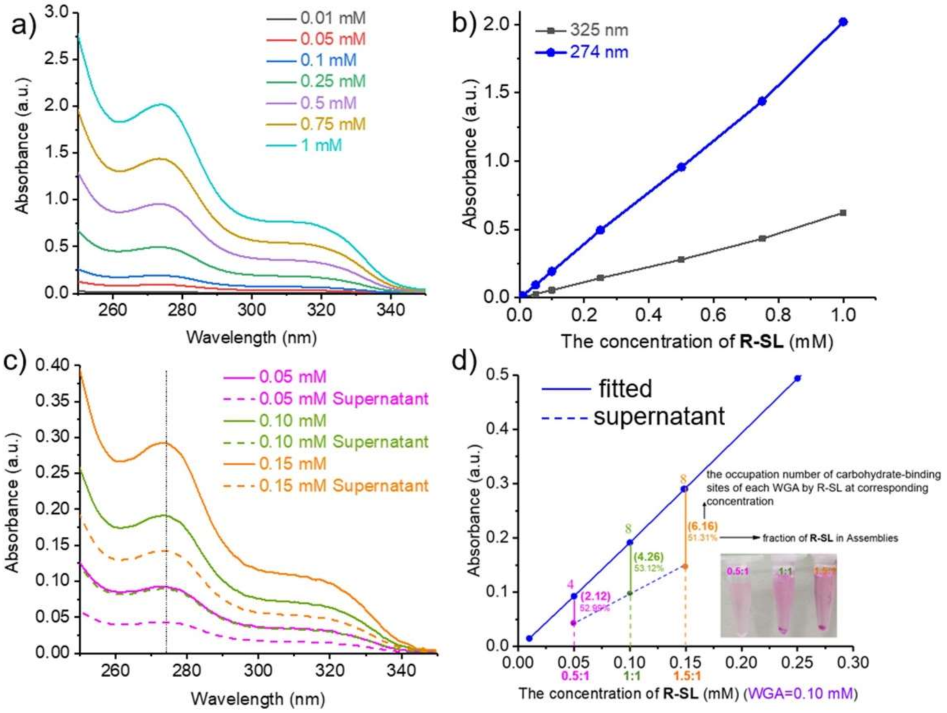

Figure S15. a) the UV-vis absorbance of **R-SL** as a function of concentration.

b) the UV-vis absorbance-Concentration curve based on data from Fig S13a.

c) the UV-vis absorbance of **R-SL** (solid line: **R-SL**, dotted line: **R-SL** in supernatant).

d) Calculated number of **R-SL** entered assemblies based on difference between fitted data (solid line) and supernatant data (dashed line).

The distribution of **R-SL** between solution and assemblies was measured as following procedure: At first, we checked the characteristic absorbance intensity at 273 nm of the self-assembly solutions with different ratios of **R-SL** to WGA such as 0.5:1, 1:1 and 1.5:1, denoted as I_1, the blue solid curve in Fig S13d therefore was obtained. Then centrifugated above-mentioned three solutions to remain supernatant, of which the characteristic absorbance peak at 273nm were checked, denoted as I_2, thereby the blue dashed line was obtained. As a result, the fraction of **R-SL** entered self-assemblies could be calculated from $(I_1-I_2)/I_1$.

11. The self-assembly behavior of R-SL/WGA = 0.5:1

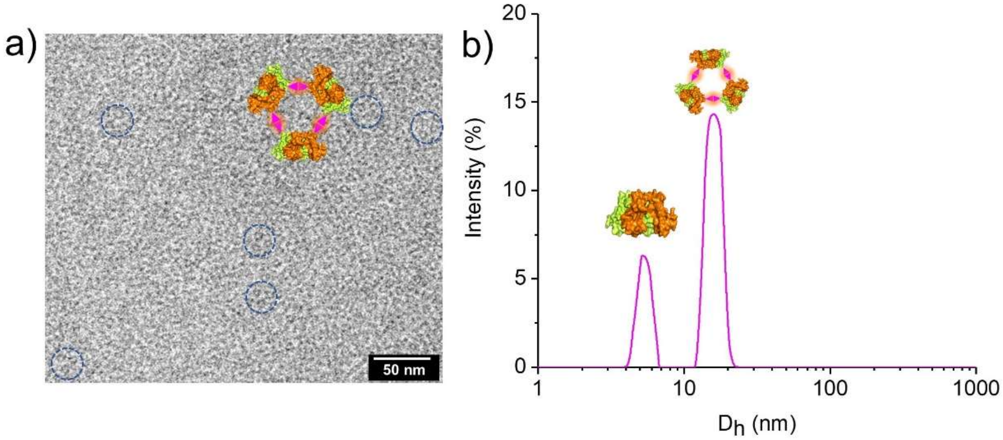

Fig S16. a) the cryo-EM image b) DLS result of **R-SL**/WGA (0.5:1) after incubation for 48 h.

12. Protein-protein Interaction

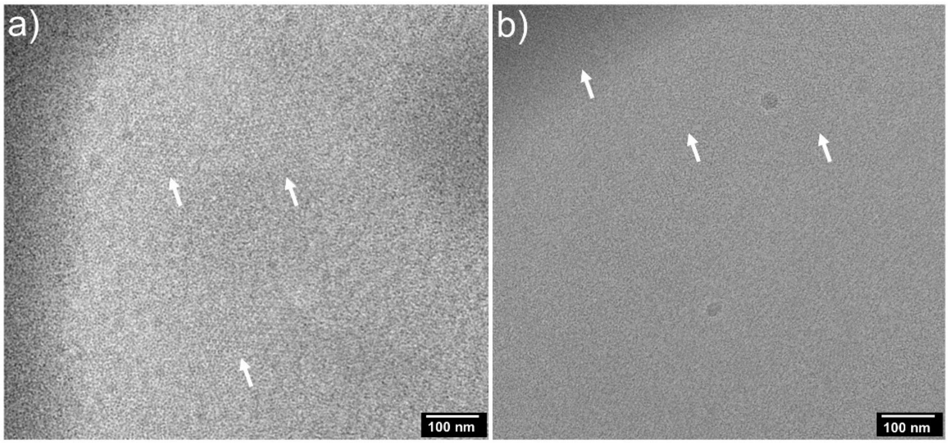

Figure S17. a, b) The Cryo-EM images of **R-SL**/WGA (1:1) after incubation for 120 h. Even if extended the incubation time to 120 h, only the 2D lattice can be observed, no 3D crystals can be found.

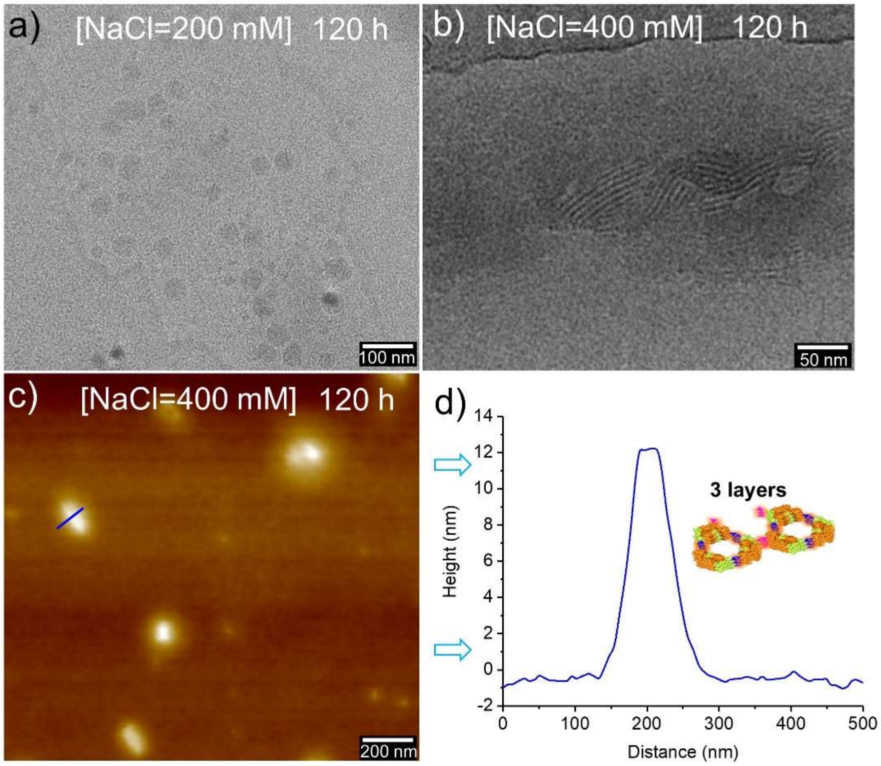

Figure S18. a, b) Cryo-EM images c) AFM height image of **R-SL**/WGA (1:1) with different NaCl concentrations. (a: 200 mM, b: 400 mM). d) the corresponding height profile of 3D crystals shown in c.

13. Characterization of 3D crystals

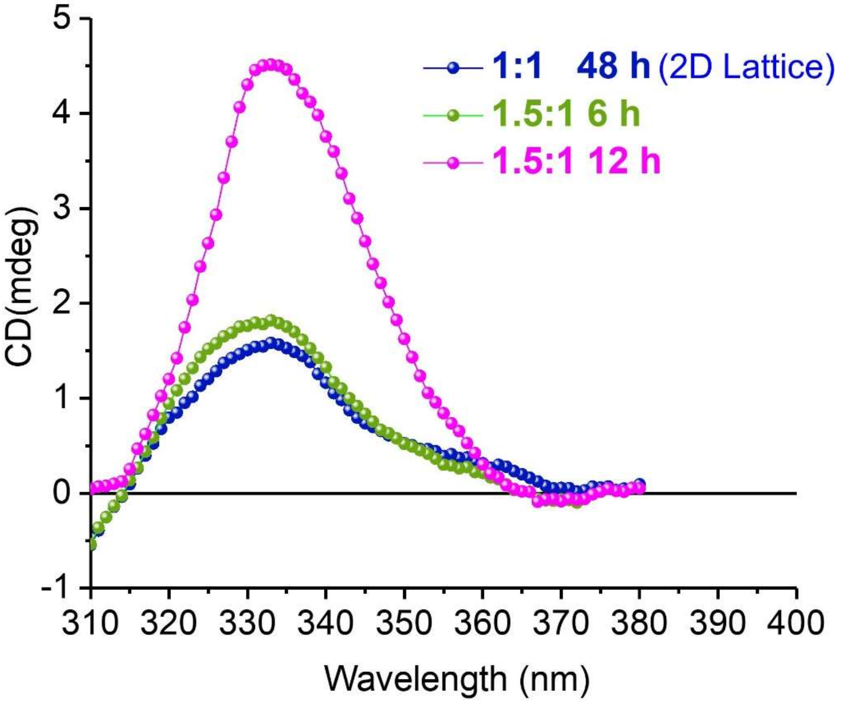

Figure S19. CD result of **R-SL**/WGA at different ratios and incubation time.

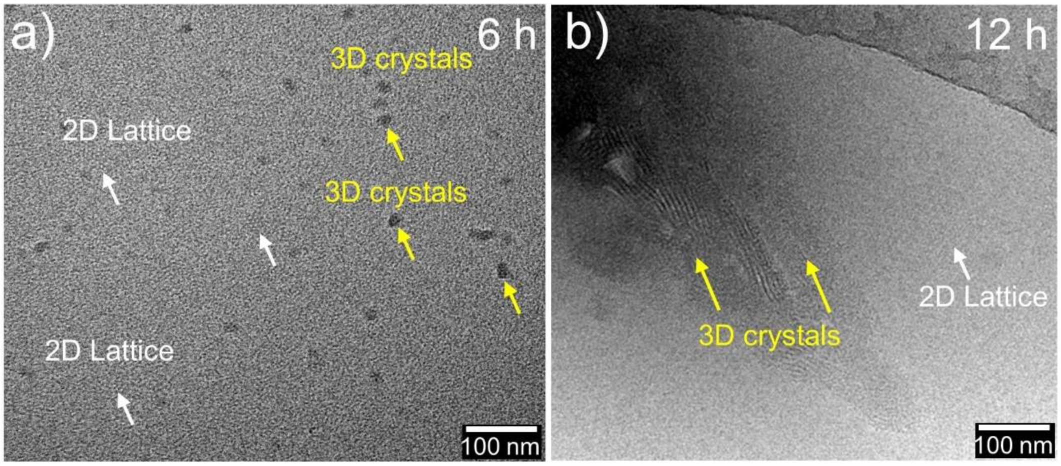

Fig S20. a, b) Cryo-EM images of self-assemblies of **R-SL**/WGA (1.5:1) after incubation for 6 h and 12 h. (white arrows: remained Pascal triangles, yellow arrows: 3D crystals)

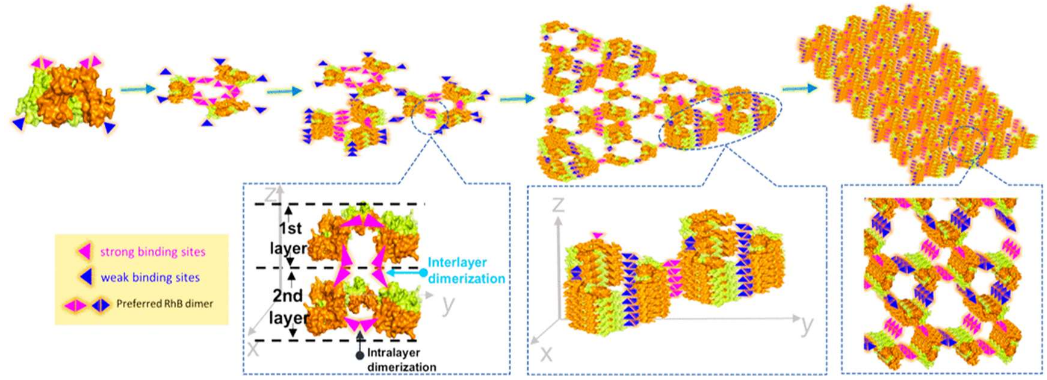

Figure S21. The proposed assembly mechanism of 3D Crystals.

14. ^{1}H and ^{13}C NMR of R-SL

Synthesis of RhB-hydrazide

To synthesize **Rhodamine B hydrazide**[15], in a 50 mL flask, Rhodamine B (0.60 g, 1.25 mmol) was dissolved in 20 mL ethanol. 8 mL (excess) hydrazine hydrate (60 %) was then added drop wise with vigorous stirring at room temperature. After the addition, the stirred mixture was heated to reflux in an air bath for 6 h. The solution changed from dark pink to light orange. Then the mixture was cooled and solvent was removed under reduced pressure. 1 M HCl (25 mL) was added to the solid in the flask to generate a clear red solution. After that, 1 M NaOH (35 mL) was added slowly with stirring until the pH of the solution reached 9~10. The resulting precipitate was filtered and washed 3 times with 15 mL water. After drying under an IR light, the reaction afforded 0.46 g Rhodamine B hydrazide (81.2 %) as pink solid. MALDI-TOF Mass Spectrum (M/z): [RhB-hydrazide]$^+$ calcd. for $C_{28}H_{33}N_4O_2$+, 457.26; found, 457.28. ^{1}H NMR (400 MHz, Chloroform-*d*) δ 8.01 – 7.90 (m, 1H), 7.57 – 7.37 (m, 1H), 7.18 – 7.08 (m, 1H), 6.53 – 6.42 (m, 2H), 6.32 (dd, J = 8.8, 2.6 Hz, 1H), 3.64 (s, 1H), 3.36 (q, J = 7.1 Hz, 4H), 1.19 (t, J = 7.0 Hz, 6H).

Synthesis of R-SL

RhB-hydrazide[16] 0 mg, 0.21 mmol) was dissolved in anhydrous methanol (2 mL) while stirring at 30 °C under a N_2 atmosphere. *Sialyllactose (2,3)* (100 mg, 0.15 mmol) and $Y(OTf)_3$ (37.1 mg, 0.06 mmol) were subsequently added into the **RhB-hydrazide** solution and the mixture was allowed to stir at 30 °C for 24 h. After completion, the mixture was concentrated and the crude product was purified using column chromatography (DCM /MeOH, 5:6, v/v) resulting in the isolation of the **R-SL** as a pink solid. Percent yield of mannose-Rh conjugate was gravimetrically determined (80%). MALDI-TOF Mass Spectrum (M/z): [R-SL] calcd. for $C_{51}H_{70}ClN_5O_{20}$, 1072.46; found, 1072.16. ^{1}H NMR (400 MHz, DMSO-d_6) δ 8.13 (d, J = 6.3 Hz, 1H), 7.59 – 7.45 (m, 1H), 7.00 (d, J = 7.0 Hz, 1H), 6.46 – 6.21 (m, 3H), 4.86 (d, J = 6.0 Hz, 1H), 4.67 (t, J = 5.5 Hz, 1H), 4.59 (d, J = 3.4 Hz, 1H), 4.19 (t, J = 5.8 Hz, 1H), 4.12 – 4.02 (m,

1H), 3.92 (dd, J = 9.9, 3.1 Hz, 1H), 3.68 (s, 1H), 3.56 (dd, J = 8.7, 4.1 Hz, 2H), 3.29 – 3.21 (m, 2H), 3.13 (t, J = 9.2 Hz, 1H), 3.04 (t, J = 9.0 Hz, 1H), 2.93 (td, J = 8.8, 2.4 Hz, 1H), 2.74 (dd, J = 11.8, 4.7 Hz, 1H), 1.90 (s, 1H), 1.36 (t, J = 11.5 Hz, 1H), 1.24 (s, 1H), 1.10 (t, J = 6.9 Hz, 5H).

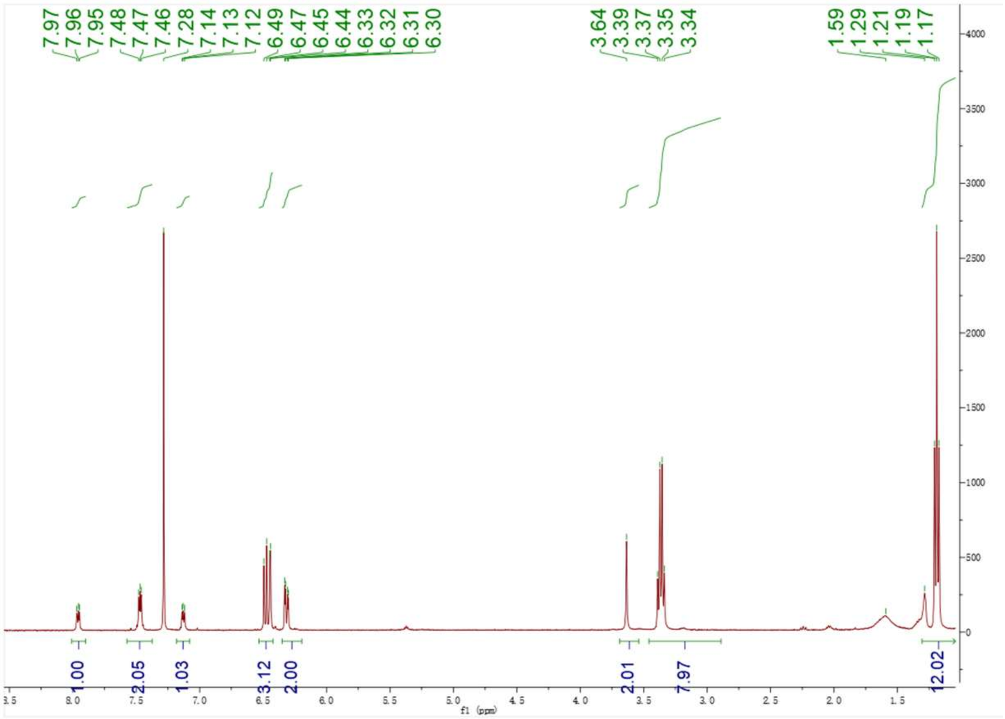

Figure S22. The ^{1}H NMR of RhB-Hydrazide in d_6-DMSO.

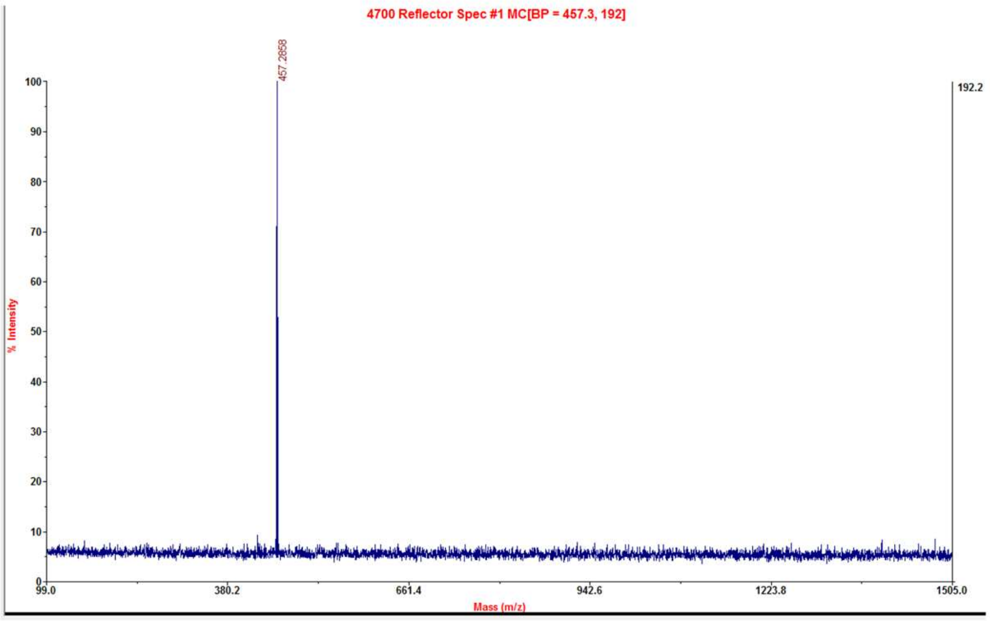

Figure S23. The Maldi-tof data of RhB-Hydrazide

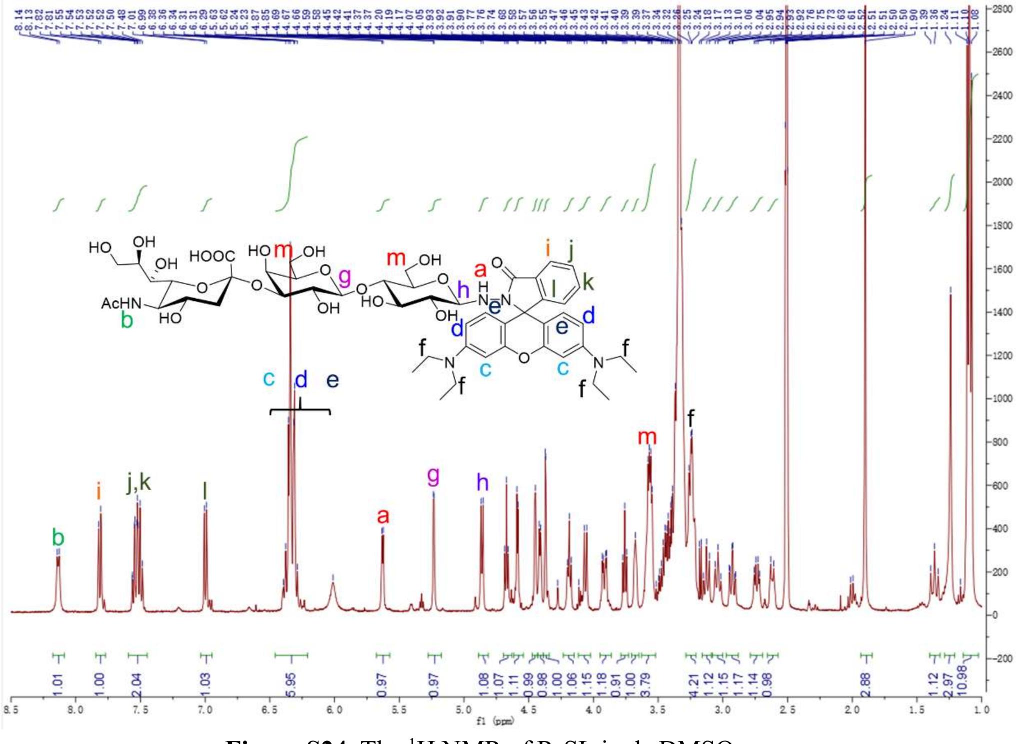

Figure S24. The ^{1}H NMR of R-SL in d_6-DMSO.

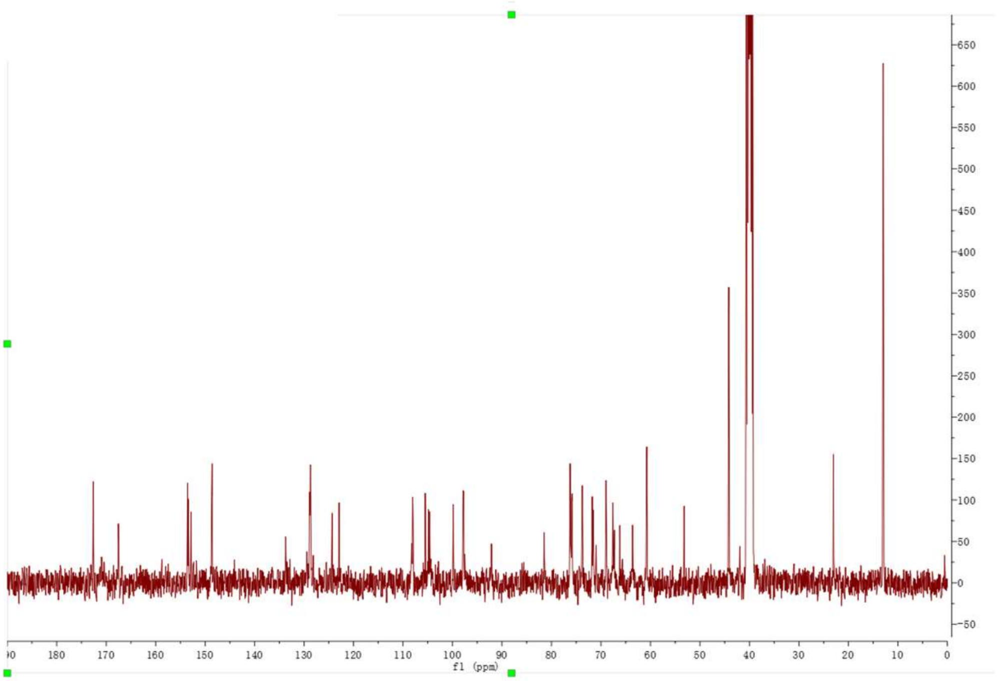

Figure S25. The ^{13}C NMR of R-SL in d_6-DMSO.

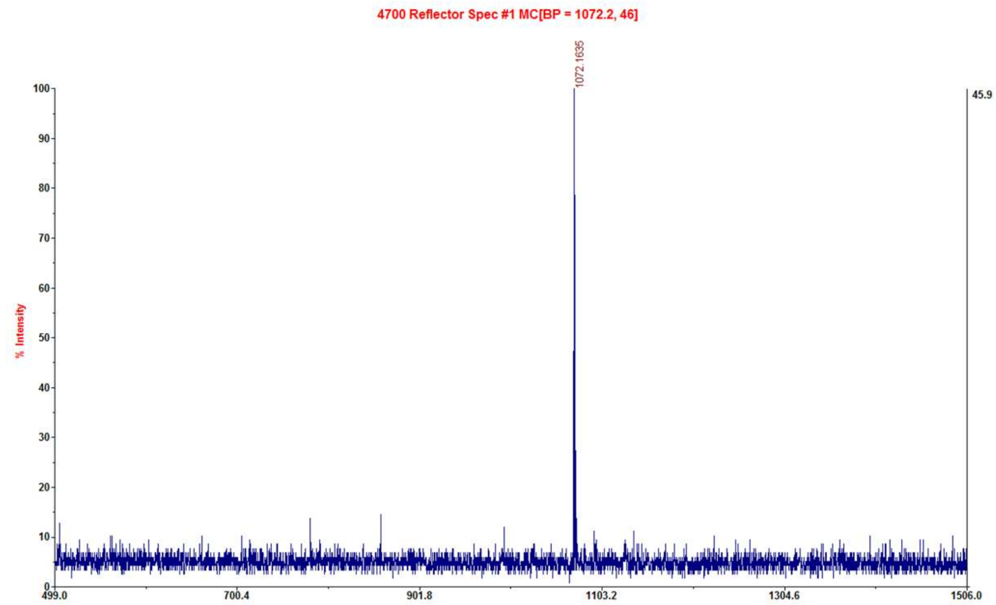

Figure S26. The Maldi-tof data of R-SL.

Reference:

1. Wright, C. S. *J. Biol. Chem.* **1992**, 267, 14345-14352.
2. Spoel, D. V. D.; Lindahl, E.; Hess, B.; Groenhof, G.; Mark, A.E.; Berendsen, H. J. C. *J. Comput. Chem.* **2005**, 26, 1701.
3. Case, D. A.; Cheatham III, T. E.; Darden, T.; Gohlke, H.; Luo, R.; Merz, K. M.; Onufriev, A.; Simmerling, C.; Wang, B.; Woods, R. *J. Computat. Chem*. **2005**, 26, 1668.
4. Jorgensen, W. L.; Madura, J. D. *J. Am. Chem. Soc.* **1983**, 105, 1407.
5. da Silva, A. W. S.; Vranken, W. F. *BMC Research Notes* 2012, 5, 367.
6. Nose, S. *J. Chem. Phys*. **1984**, 81, 511.
7. Hoover, W. *Phys. Rev. A*. **1985**, 31, 1695.
8. Essmann, U.; Perera, L.; Berkowitz, M. L.; Darden, T.; Lee, H.; Pedersen, L. G. *J. Chem. Phys*. **1995**, 103, 8577.
9. T. Schneider, E. Stoll, *Phys. Rev. B*. **1978**, 17, 1302.
10. H. M. Ding, Y. Q. Ma, *Biomaterials*. **2014**, 35, 8703-8710.
11. Tagliazucchi M, Peleg O, Kroger M, Rabin Y, Szleifer I. *Proc Natl Acad Sci U S A* **2013**;110:3363-3368.
12. Q. L. Lei, J. W. Feng, H. M. Ding, C. L. Ren, Y. Q. Ma, *ACS Macro Lett*. **2015**, 4, 1033.
13. G. Yang, R. T. Hu, H. M. Ding, Z. Kochovski, S. L. Mei, Y. Lu, Y. Q. Ma, G. S. Chen, M. Jiang, *Mater. Chem. Front*. **2018**, 2, 1642-1646.
14. S. Plimpton, *J. Comput. Phys*. **1995**, 117, 1.
15. Sikdar, Anindita, et al. *Journal of fluorescence.* **2013***,* 23.3, 495-501.
16. Trungkathan, Samon, et al. *Journal of Applied Polymer Science.* **2014**, 131, 40012 (1−9).
